# Morphometric micro-CT study of contralateral mandibular incisors

**DOI:** 10.1007/s00784-023-05419-y

**Published:** 2023-12-26

**Authors:** Usame Sevgi, Gaute Floer Johnsen, Badra Hussain, Lucila Piasecki, Liebert Parreiras Nogueira, Håvard Jostein Haugen

**Affiliations:** 1 DF Dental Group, Istanbul, Turkey; 2 Department of Biomaterials, Institute of Clinical Dentistry, Faculty of Dentistry, University of Oslo, Oslo, Norway; 3 Tanngarden AS, Sørumsand, Norway; 4 Oral Research Laboratory Institute of Clinical Dentistry, Faculty of Dentistry, University of Oslo, Oslo, Norway; 5 Department of Periodontics and Endodontics, University at Buffalo, Buffalo, NY USA

**Keywords:** Morphological symmetry, Contralateral incisors, Micro-CT, Bilateral anatomy, Comparison, Bilateral symmetry, Standardization

## Abstract

**Objectives:**

This study aimed to determine the degree of similarity and symmetry in the anatomy of contralateral mandibular incisors. Three-dimensional (3D) models of extracted teeth were obtained from microtomography (micro-CT) scans. Qualitative and quantitative assessments of the morphology and comparison of contralateral pairs were made. The null hypothesis was that contralateral mandibular incisors could not be considered identical in simple morphometric measurements.

**Methods:**

Sixty pairs of mandibular incisors were extracted from 30 patients and scanned with micro-CT. Virtual models of the cemento-enamel junction to the root apex were rendered. Parameters such as length, canal width, dentinal thicknesses, tortuosity, centerline length, accessory canals, root canal configurations, and root canal orifice cross-sections were used to compare the teeth. Width and thickness comparisons between paired teeth in the same individual were made by paired *t*-test (Wilcoxon signed-rank test for variables not normally distributed). An online randomization tool generated randomized pairs (independent of the individual/patient). Subsequently, an unpaired *t*-test (or Mann–Whitney *U* test for non-normally distributed parameters) and a correlation analysis were conducted. Canal configurations were classified according to preexisting classification schemes. The number and location of accessory canals and apical foramina were registered and compared.

**Results:**

Utilizing advanced imaging techniques and quantitative analyses, our study establishes that contralateral mandibular incisors exhibit a remarkable degree of symmetry in multiple morphological parameters, including length, canal width, and dentinal thicknesses. The apical third showed a high degree of inter-variability for the contralateral pairs. The rigorous statistical analysis of the normalized parameters by *Z*-score showed no statistically significant differences between the contralateral mandibular incisors. Comparisons between central and lateral teeth revealed differences in root length but no significant disparity in the distribution of accessory canals. Central teeth, on average, were longer, while accessory canals were distributed relatively evenly between central and lateral teeth.

**Conclusions:**

The findings of this study further establish the significant similarities between contralateral mandibular incisors, reinforcing their suitability as a reliable substrate for root canal comparison studies.

**Clinical relevance:**

The absence of statistically significant differences between contralateral pairs in normalized parameters underscores their potential as a reliable reference point for root canal comparison studies in clinical dentistry. Furthermore, our findings emphasize the importance of individualized treatment planning, considering the natural symmetry in mandibular incisors to enhance clinical decision-making. This research contributes valuable insights to the field of endodontics, offering a standardized approach to sample selection and enriching the understanding of dental anatomy.

## Introduction

Preparation, irrigation, and obturation comprise the essential steps of the therapeutic triad of root canal treatment (RCT) [1, 2]. Successful outcomes from RCT should be expected when each step is executed with the highest degree of precision under the auspices of proper aseptic protocols. With this triad in mind, endodontic research has tried to enhance and evaluate each step using comparison studies considering, for example, shaping [3–7], leakage [8–11], and cleaning ability [12–14].

Several papers have called for standardization in endodontic comparison studies [15–20]. Moreover, creating balanced experimental groups is of the utmost importance for comparative studies in the endodontic field to secure the reliability and reproducibility of the findings.

For decades, human teeth were used for laboratory research on root fillings without considering the bias caused by the anatomical heterogeneity of the samples [21]. The extracted human tooth has played a vital role in dental research as its physicochemical and morphological features cannot be truly reproduced. Contralateral teeth have long been used for research purposes because they are similar in anatomy and physical characteristics [15, 22–25].

A recent study by Johnsen et al. [26] demonstrated high similarity between contralateral mandibular incisors (CMI) when considering their three-dimensional geometric morphometric micro-computed tomography (micro-CT) scans. This study only analyzed the symmetry between the incisors and did not compare specific anatomical characteristics.

This study aimed to investigate contralateral incisors’ morphometric properties and anatomic parameters using advanced imaging techniques such as micro-CT and quantitative analyses. The null hypothesis is that contralateral mandibular incisors do not exhibit similar parameters: length, canal width, dentinal thickness, tortuosity, centerline length, accessory canals, root canal configurations, and root canal orifices.

## Materials and methods

### Sample selection

160 permanent mandibular incisors from 40 volunteer patients were extracted for reasons unrelated to this study. All methods were carried out in accordance with relevant guidelines and regulations, and informed consent was obtained from all subjects. This study was approved by the local Institutional Review Board (no. 00003080), Department of Periodontics and Endodontics, University at Buffalo, New York, USA. Teeth were evaluated under magnification, and radiographs were taken from coronal and sagittal views. Exclusion criteria included cracked or fractured roots, calcified canals, immature apices, resorptive defects, extensive caries, filling materials, and/or previous root canal access. After applying these criteria, 120 sound mandibular incisors obtained from 30 patients were included in this study. The four incisors of each patient were examined, identified, aligned, and fixed in a Styrofoam jig according to their respective position in the arch for easy recognition. Samples were kept in plastic containers in 100% humidity. The same set of incisors was used in a previous study where the aim was to investigate the matching symmetry of contralateral mandibular incisors [26].

### Micro-computed tomography

The samples were scanned with a Multiscale (SkyScan 2211 Multiscale X-ray Micro-CT System, Bruker micro-CT, Kontich, Belgium) equipment with a 20–190 kV tungsten X-ray source with a detection system consisting of a 3-megapixel 1920 × 1536 pixels flat panel detector. Contralateral mandibular incisors were scanned at 65 kV, 55 μA, and 120 ms, with an aluminum filter of 0.5-um thickness. The rotation step was set to 0.79°, using frame averaging of 3, and overall scans were taken over 360°. Each scan achieved a voxel size of 15 μm using the flat panel detector with a scan duration of 13 min. Projections were reconstructed using the default software of the equipment, NRecon (version 1.7.4.6), with the following parameters: smoothing factor: 0; ring artifact correction: 8; filter cutoff relative to Nyquist frequency: 100; filter type description: hamming (alpha = 0.54), undersampling factor = 1, threshold for defect pixel mask (%): 0; and beam hardening correction (%): 50. 2D/3D image registration was made with DataViewer (Bruker MicroCT, version 1.5.2.4) and analyzed with CTAn (Bruker micro-CT, Kontich, Belgium, version 1.18.4.0). After scanning, the teeth were stored in a 70% EtOH (Oslo, Norway) humidor and kept in a cold room at a constant 4 °C. This allowed for rescanning of samples if needed. The teeth were never physically contacted with the ethanol bath, and the evaporated ethanol was topped off regularly.

### Image processing and registration

CTan (Bruker micro-CT, Kontich, Belgium, version 1.18.4.0) was used to investigate different structures of the teeth separately by applying various thresholds to differentiate components and extract the geometry. Then CTvox (Bruker micro-CT, version 3.3.1) was used for presenting data together and for the measurements. Subsequently, Avizo (version 2022.2, Thermo Fisher Scientific, Waltham, Massachusetts, USA) was utilized for centerline determination, tortuosity, and length measurements. Within CTvox and Avizo, the integrated 3D image data was meticulously processed and visualized, then facilitated the creation of comprehensive 3D representations of the specimens or structures under investigation. This visualization allowed us to obtain a detailed and clear understanding of the internal and external features of the incisors. The software packages provided an array of measurement tools and functionalities crucial for precise and reliable quantitative analyses, which were employed to obtain precise measurements of various parameters.

#### Centerline determination and length measurements

The centerline algorithm within Avizo was then applied to create a precise centerline representation of the root canal. The centerline algorithm functions by identifying key points or voxels within the 3D volume data that lie along the center of the structure. It achieves this by tracing a path through the volume, typically following the intensity gradient, and identifying the points that represent the central axis. This process ensures that the centerline accurately represents the morphology and geometry of the structure.

#### Measurement of root canal parameters

**Fig. 1:**
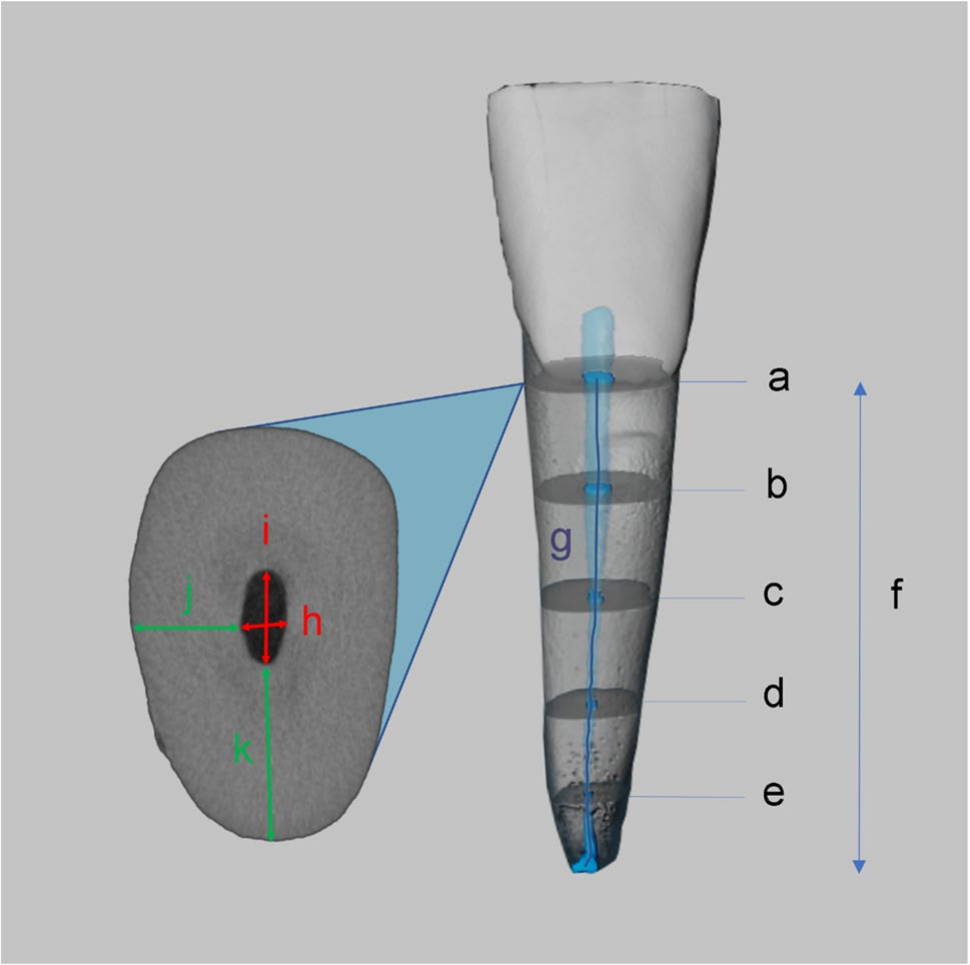
Linear measurements of mandibular contralateral incisors. Length (CEJ to apex [f] and the centerline of the root canal [g]), root canal widths with the longest distance in the mesiodistal and labiolingual directions (h, i), and longest dentinal thicknesses in two directions (j, k) of each intersection. Intersections were chosen as CEJ (a), 2 mm before apex (e), and each quarter way between them (b, c, d)

Once the centerline was established, the Avizo software allowed for length measurements to be made along this centerline. The software utilized its measurement tools to calculate distances between specific points or landmarks along the centerline, enabling precise length measurements from cemento-enamel junction (CEJ) to apex, and tortuosity between this centerline and the linear length of the root canal. Various linear measurements of contralateral incisors were undertaken for analysis (Fig. 1a–f) with this Avizo software. For reference, CEJ was defined as the final coronal cross-section with visible enamel. The apex was defined as the last apical cross-section displaying visible dentin. The CEJ was the first cross-section with visible enamel in the cervical line, and the apex was the previous final apical cross-section with visible cementum. Then, length measurements (Fig. 1f and g) were performed from the cemento-enamel junction (CEJ) to the apex following the long axis (Fig. 1f) and the centerline of the root canal (Fig. 1g). The centerline was also used to define the tortuosity of the canals. The root canal widths (Fig. 1h and i) were measured using CTan software as the longest distance in the mesiodistal and the labiolingual directions at five levels (Fig. 1a–e) at the CEJ and 2.0 mm before the apex, as well as a fourth of the distance between them. The dentinal thicknesses (Fig. 1j and k) were measured as the shortest distances in the mesiodistal and the labiolingual directions at the previously mentioned levels.

In the course of this study, the number and shape of root canal orifices at the CEJ level, root canal configurations, and accessory canals were either described or measured. These parameters were considered to characterize the examined incisors’ anatomical features comprehensively.

#### Root canal orifice characterization and classification

The type of root canal orifices was noted and classified in each sample’s CEJ level section as CTan software presented.

#### Root canal configuration and classification

The 3D models of the mandibular anterior teeth were analyzed and classified according to their root canal configurations according to Vertucci’s classification [27]. The 2D images were examined slice by slice using CTan software to have the accessory canals identified and classified [28]. In addition, the first coronal cross-section showing an accessory canal was used as a reference to record their vertical position and proportional distribution.

### Statistical analysis (canal width, dentinal thickness, canal length, and tortuosity)

For the parameters normally distributed, two-way ANOVA was used, and Wilcoxon signed-rank test was used for the variables that were not normally distributed (buccolingual canal width in the CEJ region, buccolingual canal width at the apical 2 mm, and dentinal thickness mesiolingual at first quarter distance from CEJ). Comparison between paired teeth in the same individual was made by paired *t*-test and Wilcoxon signed-rank test for variables not normally distributed, which was the width and thicknesses. An online randomizer performed randomization to create random pairs (independent of the individual/patient) (https://www.randomizer.org/). The unpaired *t*-test was conducted (Mann–Whitney *U* for the parameters not normally distributed). In addition, regression analysis was performed to evaluate the influence of the variables on the dependent outcome decided to look at.

Furthermore, a correlation analysis between the teeth was done. The scale (for the variables) is considered continuous; thus, the Pearson correlation was used for the correlation analysis. The results were interpreted as follows: no correlation if *r*_*p*_ < 0.3, correlation if 0.3 < *r*_*p*_ < 0.5, and strong correlation if 0.5 < *r*_*p*_ < 1. The significance level was set to *P* < 0.05. A negative *r*_*p*_ indicated a negative correlation, while a positive *r*_*p*_ indicated a positive correlation. All statistical analysis was performed with the software STATA 12 (Stata Corporation, College Station, TX, USA), and a *p* value below 0.05 was considered significant.

## Results

### Canal width, dentinal thickness, canal length, and tortuosity

#### Canal width

Contralateral pairs in the same individual and random individuals did not have statistically significant differences in the canal width, where none of the variables showed any statistically significant differences.

There are varying findings regarding ipsilateral. The ipsilateral in the left quadrant did have 3 out of 10 variables with statistically significant differences for random pairs (*p* < 0.05) and 5 out of 10 variables in the same individual (*p* < 0.05). Ipsilateral in the right quadrant has 1/10 statistically significant variables for random pairs (*p* < 0.5) and 4/10 for paired (*p* < 0.05).

Regarding the central in the left quadrant compared to the lateral in the right quadrant, 3/10 variables in the paired dataset showed a statistically significant difference (*p* < 0.05). And for the randomized dataset, there were 2/10 that led to statistically significant differences (*p* < 0.05).

In addition, comparing the central in the right quadrant to the lateral in the left quadrant, 4/10 variables in the paired dataset showed a statistically significant difference (*p* < 0.05) and 2/10 for the randomized pairs (*p* < 0.05).

#### Dentinal thickness

Contralateral pairs in the same individual and random individuals did not have statistically significant differences in the dentinal thickness (none of the variables showed statistically significant differences). Regarding ipsilateral, there were variables with statistically significant differences between the teeth. For example, ipsilateral in the left quadrant did have 3/10 variables with statistically significant differences for random pairs (*p* < 0.05) and 4/10 for paired (same individual), *p* < 0.05. Ipsilaterals in the right quadrant had 3/10 variables with statistically significant differences for random pairs (*p* < 0.05) and 3/10 for paired data (*p* < 0.05).

Regarding the central in the left quadrant compared to the lateral in the right quadrant, 3/10 variables in both the paired and the randomized dataset showed statistically significant differences (*p* < 0.05). The same finding was observed for the central in the right quadrant compared to the lateral in the left quadrant; 3/10 variables showed statistically significant values for both the randomized and paired datasets (*p* < 0.05).

### CEJ-apex length and tortuosity

#### Correlation for CEJ-apex length and tortuosity

Centerline length is correlated between all four teeth, and CEJ-apex length is correlated between all four teeth, with the following correlation coefficients: 42 and 32 (*r* = 0.94, *p* < 0.001), 41 and 31 (*r* = 0.94, *p* < 0.001), 42 and 41 (*r* = 0.81, *p* < 0.001), 42 and 31 (*r* = 0.82, *p* < 0.001), 32 and 31 (*r* = 0.77, *p* < 0.001), and 32 and 41 (*r* = 0.78, *p* < 0.001).

Tortuosity does not have the exact correlation as the two aforementioned parameters; central (41 and 31, *r* = 0.49, *p* < 0.01) and lateral contralateral teeth (32 and 42, *r* = 0.43, *p* < 0.02) are correlated regarding tortuosity. And the lateral in the left quadrant is correlated to both centrals (32 and 41, *r* = 0.63, *p* < 0.001; 32 and 31, *r* = 0.39, *p* < 0.03).

#### Root canal orifice shapes and types

**Fig. 2:**
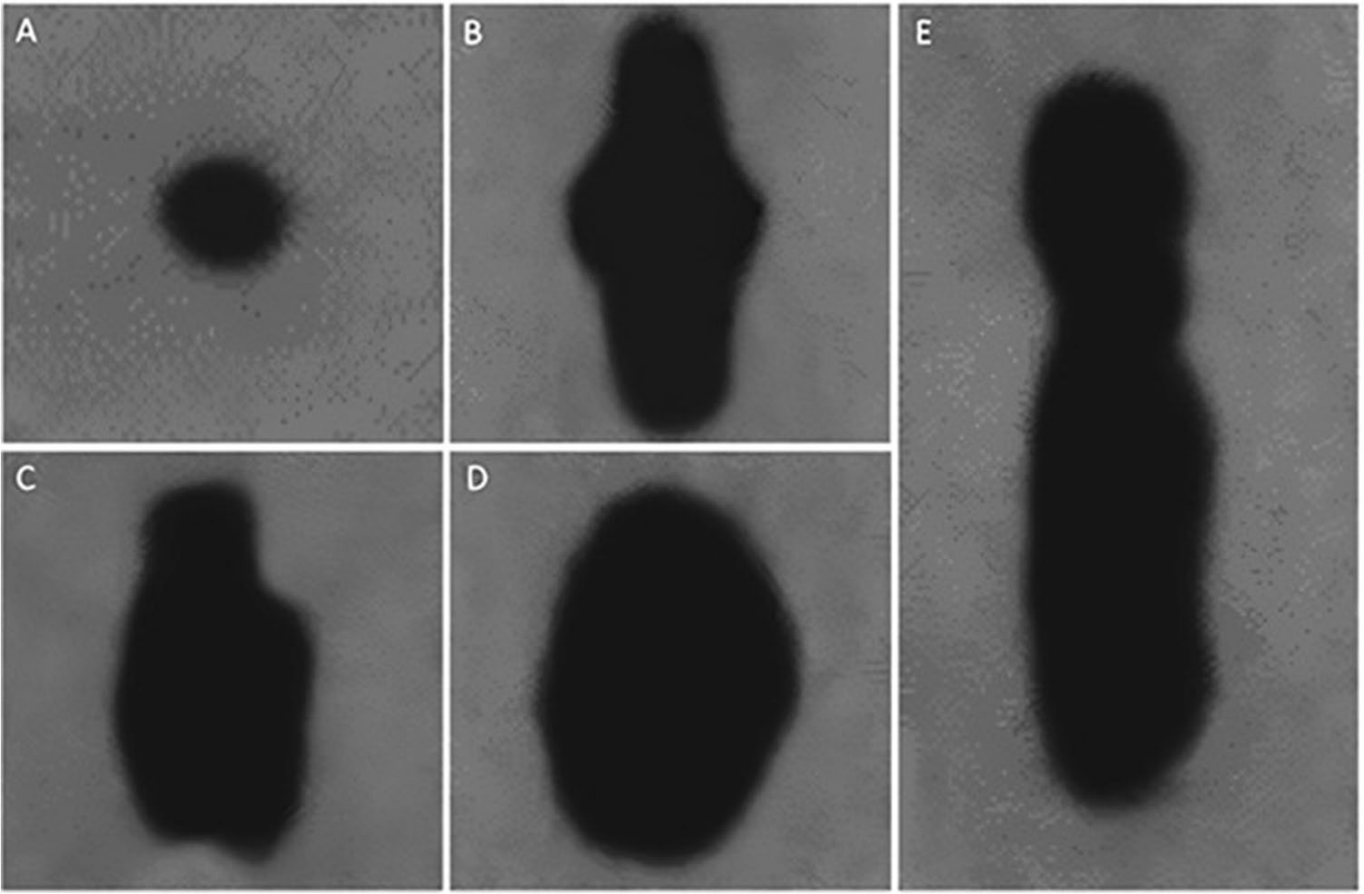
Different root canal orifice shapes found in mandibular incisors at cemento-enamel junction level: circular (**A**), fish (**B**), bowling pin (**C**), oval (**D**), and hourglass (**E**)

Five different types of orifice shapes were found at the CEJ level (Fig. 2): hourglass (56.67%), oval (26.66%), fish (10%), circular (4.17%), and bowling pin (2.50%). 52 of 60 pairs (74.28%) had the same orifice shape, while the similarity in CMI was found in 20 of 30 patients. Eight pairs showed different properties from each other in terms of orifice shapes (Table 1).

**Table 1:** Distribution of root canal orifice shapes at CEJ level found in mandibular incisors

Root canal orifice shapes at cemento-enamel junction level	Amount (%)	Similarity		
		Quadruple match	Pair match	Single
Hourglass	68 (56.67%)	56 (82.36%)	8 (11.76%)	4 (5.88%)
Oval	32 (26.66%)	20 (62.50%)	8 (25%)	4 (12.50%)
Fish	12 (10%)	0 (0%)	6 (50%)	6 (50%)
Circular	5 (4.17%)	4 (80%)	0 (0%)	1 (20%)
Bowling pin	3 (2.50%)	0 (0%)	2 (66.67%)	1 (33.33%)

### Root canal configurations

**Fig. 3:**
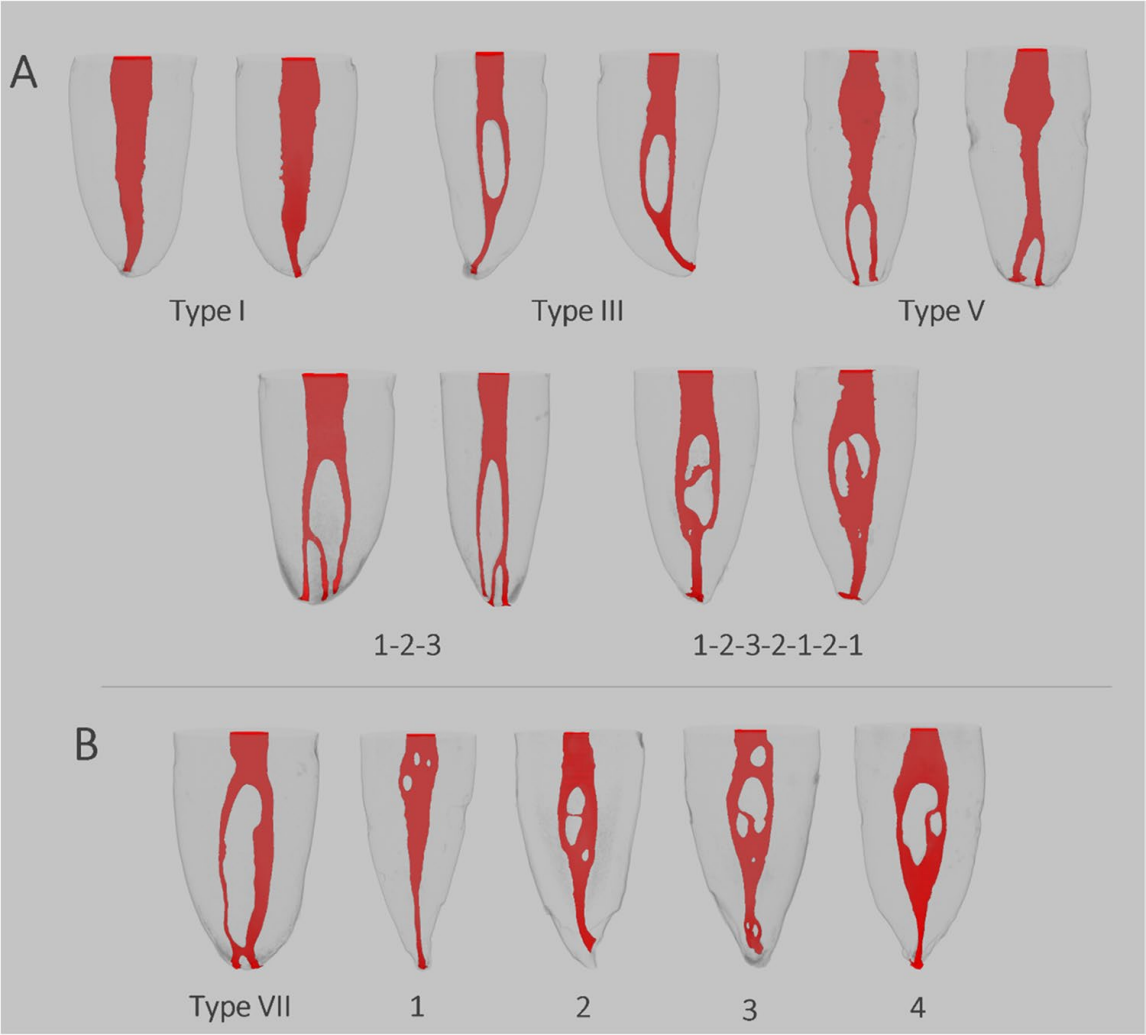
Virtual models of the roots. (**A**) Root canal configurations found in contralateral relation: type I (40 pairs), type III (8 pairs), type V (1 pair), 1–2-3 configuration (1 pair), and 1–2-3–2-1–2-1 configuration (1 pair). (**B**) Additional root canal configurations found in mandibular incisors: type VII in one mandibular right central, (1) 1–2-3–1-2–1 configuration in one mandibular right lateral, (2) 1–2-1–2-1–2-1 configuration in one mandibular right central, (3) 1–2-1–2-3–2-1–2-1–2-3–2-1 configuration in one mandibular left central, and (4) 1–2-3–2-1 configuration found in two samples. Note that (1), (2), and (3) were obtained from the same patient

There were ten different root canal configurations (Fig. 3). The most common types in ascending order were Vertucci type I (70.83%), type III (17,50%), type V (2,50%), and type VII (0.83%). Several configurations (8.34%) could not be classified according to Vertucci’s original scheme of eight (Fig. 3). The relation of CMI was found to have a quadruple similarity in Vertucci type I in 17 (56.67%) patients, quadruple similarity in Vertucci type III in 1 (3.33%) patient, contralateral similarity within pairs in 4 (13.34%) patients, triple match in 6 (20%) patients, one pair match in CMI in 1 (3.33%) patient (Table 2). Contralateral pairs were found to be matched in Vertucci type I in 18 (30%) central and 22 (36.67%) lateral pairs, Vertucci type III in 6 (10%) central and 2 (3.33%) lateral pairs, Vertucci type V in 1 (1.67%) lateral pair, 1–2-3 configuration in 1 (1.67%) central pair, and 1–2-3–2-1–2-1 configuration in 1 (1.67%) lateral pair (Table 3). A total of 5 (8.33%) central and 4 (6.67%) lateral pairs did not have identical root canal configurations.

**Table 2:**
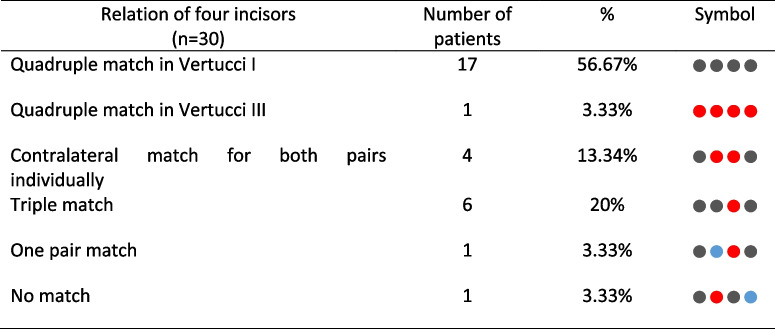
Distribution of root canal configurations found in CMI

**Table 3:** Distribution of Vertucci types over pairs and their occurrence rate in central and lateral teeth

Relation of contralateral pairs		Number of pairs (%)	Tooth type	Amount by pair type
Vertucci I		40 (66.66%)	Central pairs	18 (30%)
			Lateral pairs	22 (36.70%)
Vertucci III		8 (13.33%)	Central pairs	6 (10%)
			Lateral pairs	2 (3.33%)
Vertucci V		1 (1.67%)	Central pairs	0
			Lateral pairs	1 (1.67%)
Unclassifiable	1–2-3	1 (1.67%)	Occurred in central pairs	1 (1.67%)
	1–2-3–2-1–2-1	1 (1.67%)	Occurred in lateral pairs	1 (1.67%)
The pairs did not match		9 (15%)	Central pairs	5 (8.33%)
			Lateral pairs	4 (6.67%)

### Accessory canals

**Fig. 4:**
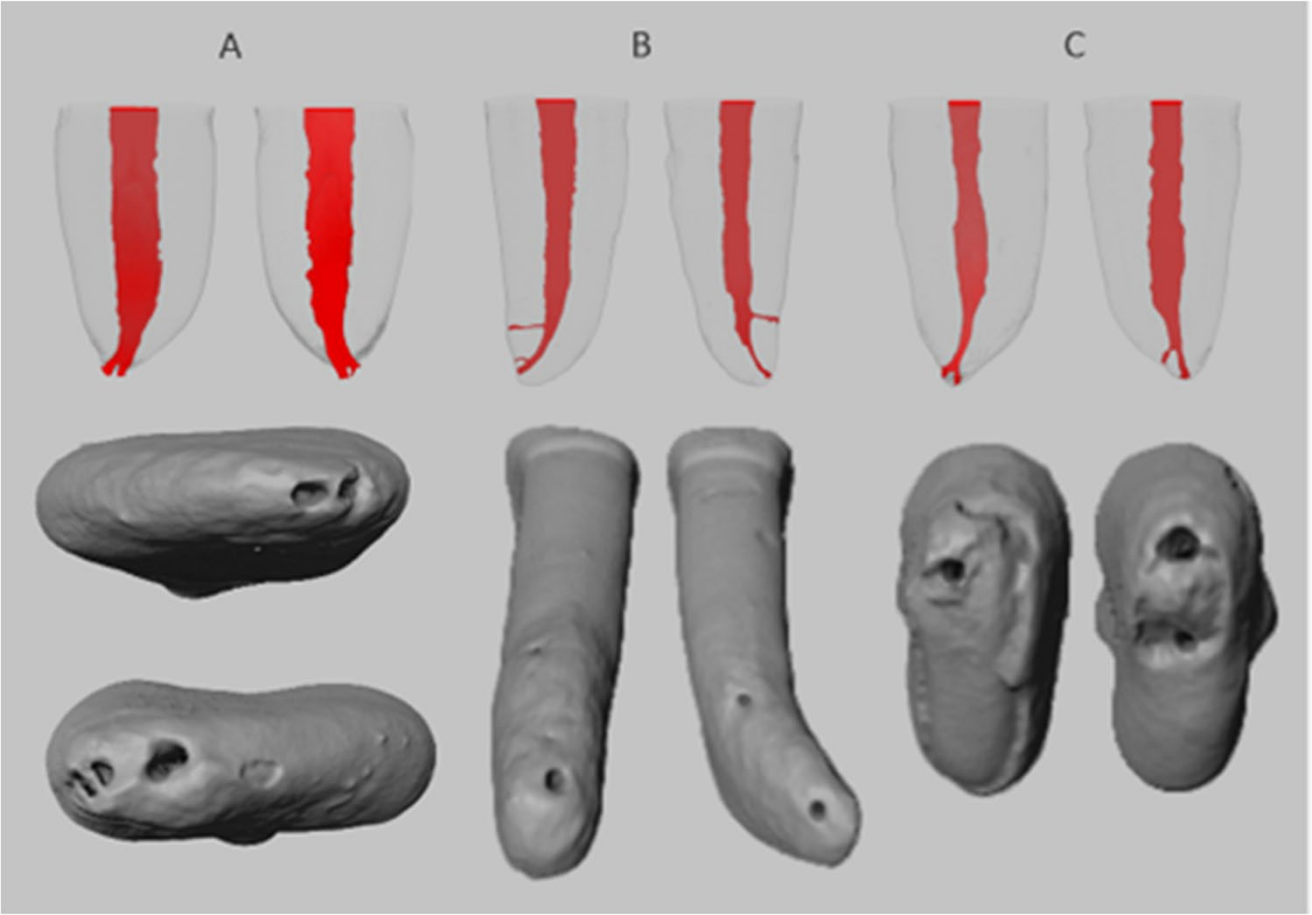
Examples of contralateral presence of accessory canals. (**A** and **C**) Contralateral pairs of mandibular central incisors. (**B**) A contralateral pair of mandibular lateral incisors. The top row displays the outline of the roots and the pulp cavity in red. The bottom row displays the apical view of the root surfaces of the same models (opaque gray), showing the apical foramina

**Fig. 5:**
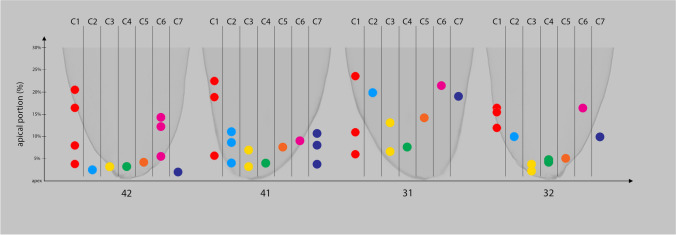
Accessory canal distribution in apical third, when seen in all CMI from the same patient: 1: mandibular right permanent lateral incisor; 2: mandibular right permanent central incisor; 3: mandibular left permanent central incisor; and 4: mandibular left permanent central incisor. C: contralateral mandibular incisors. Each color shows different contralateral teeth sets of mandibular incisors obtained from different individuals

**Fig. 6:**
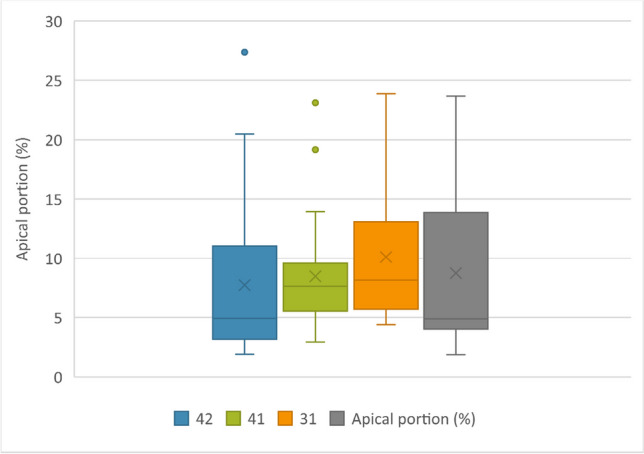
Box plots of the overall vertical distribution of accessory canals: 42: mandibular right permanent lateral incisors; 41: mandibular right permanent central incisors; 31: mandibular left permanent central incisors; and 32: mandibular left permanent central incisors

63 teeth (52.50%) were found to have accessory canal formation. The accessory canals were found in the apical third (95.83%) and the middle third (4.17%). No accessory canals were found in the coronal third. Forty-four (22 pairs) out of 63 teeth (69.84%) presented accessory canals contralaterally, and 12 of these pairs (54.55%) had the same number of accessory canals. Among the 57 teeth with no accessory canal, 36 (18 pairs) were contralateral pairs (63.15%) (Fig. 4). A total of 15 (12.50%) teeth presented more than one accessory canal, and 6 were found to be paired. Only one tooth (0.83%) showed an apical delta. Concerning the symmetry between four incisors, 28 teeth (44.44%) from 7 CMI had at least one accessory canal among 63 samples (Fig. 5). Also, 20 teeth (35.09%) from 5 CMI, out of 57 teeth, had no accessory canal formation. The proportional location of accessory canals in the apical third had a mean value of 4.15%, 7.64%, 8.13%, and 4.88% of the portion of the root in left lateral, left central, right central, and right lateral mandibular incisors, respectively (Fig. 6).

## Discussion

Contralateral mandibular incisors exhibit similarities that suggest their potential for being considered identical [26]. This present study supports previous findings and strengthens the evidence for using contralateral incisors as a substrate for root canal comparison studies. The authors conducted an in-depth analysis of contralateral mandibular incisors, focusing on various morphological parameters. The investigation encompassed root canal configurations, accessory canal formation, and their distribution across central and lateral incisors. The research yielded several noteworthy findings, which have significant implications for the field of endodontics.

Primarily, the study revealed a high degree of similarity in multiple morphological parameters between contralateral mandibular incisors. Parameters such as length, canal width, and dentinal thicknesses exhibited remarkable similarity, with no statistically significant differences observed in normalized parameters, as indicated by *Z*-score analysis. This finding underscores the potential use of unaffected contralateral mandibular incisors as a reliable reference point in root canal comparison studies, offering valuable support for dental professionals in accurate treatment planning and execution of endodontic procedures.

Only a few studies have evaluated the similarity of the internal anatomy between contralateral mandibular incisors. Previous CBCT studies [29–33] lacked complete quantitative and qualitative analyses. This imaging method’s low resolution and slice thickness limitations probably hindered detailed evaluations. Compared to micro-CT, CBCT has a larger field of view [34, 35] and larger voxel sizes [36–38], thus presenting limitations in detecting complex anatomical configurations [39], explaining the contrasting results shown in the literature. Therefore, micro-CT remains the gold standard for studying dental hard tissues [40].

Johnsen et al. previously investigated the symmetry of contralateral premolars [17, 18]. They concluded that contralateral premolars could be considered mirror images in several morphological, morphometric, and linear measurements, except for the apical regions, which did not display matching symmetry. A more recent study by Johnsen et al. [26] on contralateral mandibular incisors showed a higher degree of matching symmetry than contralateral premolars [17, 18]. The present study further reinforces the concept that contralateral mandibular incisors exhibit a high degree of symmetry. Additionally, they demonstrated a lower frequency and number of accessory canals when compared to premolars. The major advantage of contralateral premolars must be that they are easier to acquire as they are often extracted during orthodontic treatment [15, 41, 42].

Previous studies have used different methods to investigate mandibular incisors’ distribution and root canal configurations. Micro-CT is considered the gold standard for studying the internal anatomy of the teeth [43] due to its ability to detect fine details and complexity of the root canal anatomy [44, 45]. While earlier studies did not report unclassifiable root canal configurations despite adequate sample size from different ethnicities [46–50], recent micro-CT studies have found a frequency of unclassifiable configurations ranging from 6 to 18% among mandibular incisors [51–55]. This study found similar results, with 8.34% of our 120 teeth being unclassifiable according to Vertucci classification [27]. However, our results may be affected by the symmetrical appearance of contralateral incisors collected from the same patient, which may have caused repetitive results. When contralateral pairs did not match in terms of root canal configurations, there was a 55.60% chance of an unclassifiable sample being presented in the pair.

In an effort to standardize our study, we utilized the novel definitions by Ahmed et al. [28] to evaluate accessory canals and the apical delta. This approach was taken considering previous discrepancies reported in root canal morphology classification.

CEJ presents a reliable landmark for clinicians to use to locate the position and floor of the pulp chamber [56]. For this reason, the CEJ was chosen as a reliable reference section for comparing several parameters in the present study. Furthermore, the configurations of root canal orifice shapes may present challenges during instrumentation and disinfection. Specifically, narrow areas and tapered shapes within the root canal system can impede instrument access, posing challenges in achieving thorough cleaning and shaping of the canal. Consequently, this may give rise to the accumulation of debris or untreated spaces within the canal.

The apical third of the root canal is considered the most complex structure of the root canal system and where internal complexities abound [57–59]. The irregularities of the apical portion are difficult to reach during instrumentation and disinfection, which may lead to failed root canal treatment [60]. In several micro-CT studies, the prevalence of apical accessory canals in mandibular anterior teeth has been reported as 13.60%, 25.90%, 28.50%, and 32% [50, 52, 55, 61]. Differently, we have found a higher occurrence rate of accessory canals in the apical third than the previous findings. However, this higher occurrence rate is likely related to the symmetry of the samples, considering that 69.84% of the apical accessory canals were identified within pairs. This observation highlights the importance of determining the origin of the teeth utilized in the investigations, as their provenance might impact the results of morphological evaluations.

One of the main goals of an apicoectomy is to resect the anatomical irregularities that orthograde root canal treatment could not disinfect while minimizing dentin sacrifice during surgery [61, 62]. To achieve optimal patient outcomes by preserving dental structure to maintain an acceptable crown-root ratio, it is essential to keep dentin sacrifice to a minimum, typically at least 3 mm. With this in mind, we investigated the location of apical irregularities in mandibular incisors. Our findings show that 95.65% of all accessory canals were located in the last 2 mm of the root. This suggests that removal of the apical 2 mm of the root may be necessary to achieve optimal outcomes for patients while minimizing dentin sacrifice during surgery, eliminating accessory canals that may harbor bacteria, and preventing complete disinfection of the root canal system. Therefore, it should be noted that this decision must be made on a case-by-case basis, considering the individual patient’s needs and circumstances.

In terms of the differences between central and lateral teeth, despite the statistically significant relation of the root length (*p* < 0.01) and centerline length (*p* < 0.05) between lateral and central teeth extracted from the same individual, lateral teeth are mostly found to be longer (%97). Accessory canals not to have a significant difference between central and lateral teeth; the overall distribution of accessory canals on central teeth was seen at 47.78%.

In 2000, Sato et al. [63] introduced the centerline algorithm, which detects the farthest voxels from the boundary field and creates a connecting line. In our study, we used the centerline algorithm to detect the central trajectory length of the root canal, which refers to the length of a curved line that runs through the center of the root canal. We calculated the tortuosity between this curved length and the linear length of the root canal, which measures the degree of curvature of the canal.

Recognizing such similarity and symmetry among contralateral tooth types will greatly aid researchers in conducting ex vivo studies with a well-balanced experimental design. This study offers an optional option for standardizing sample selection, minimizing biases and potential confounding factors, and ultimately increasing the accuracy and reliability of comparative studies in the field of Endodontics.

## Conclusion

In summary, this research provides compelling evidence to reject the null hypothesis, confirming that contralateral mandibular incisors exhibit high similarity in multiple morphological parameters, such as length, canal width, and dentinal thicknesses. The absence of statistically significant differences between the contralateral pairs in normalized parameters is particularly noteworthy, as indicated by *Z*-score analysis. The only notable exception was observed in the apical third, where inter-variability was evident.

The implications of these findings extend beyond mere academic interest. Clinically, the results substantiate using unaffected contralateral mandibular incisors as a reliable reference point for root canal comparison studies. This stands to aid dental professionals in both accurate treatment planning and effective execution of endodontic procedures. The study’s implications extend to standardizing sample selection, reducing biases, and enhancing the accuracy and reliability of comparative studies in dental research. Moreover, a nuanced understanding of the natural symmetry in mandibular incisors may be invaluable for clinicians, enriching their grasp of dental anatomy and enhancing their clinical decision-making capabilities in various dental procedures.

While this study substantially contributes to the knowledge concerning the anatomical symmetry of contralateral mandibular incisors, future research could explore the clinical implications of the observed inter-variability in the apical third. Longitudinal studies might also offer insights into how these morphological characteristics change over time and how such changes could impact endodontic success rates.

In conclusion, our findings corroborate the substantial morphological similarity between contralateral mandibular incisors and underscore their relevance in clinical dentistry, particularly in endodontics.
